# Clinicopathologic, molecular, and prognostic implications of the loss of EPCAM expression in colorectal carcinoma

**DOI:** 10.18632/oncotarget.5618

**Published:** 2015-10-30

**Authors:** Jung Ho Kim, Jeong Mo Bae, Young Seok Song, Nam-Yun Cho, Hye Seung Lee, Gyeong Hoon Kang

**Affiliations:** ^1^ Department of Pathology, SMG-SNU Boramae Medical Center, Seoul, Korea; ^2^ Department of Pathology, Seoul National University College of Medicine, Seoul, Korea; ^3^ Laboratory of Epigenetics, Cancer Research Institute, Seoul National University College of Medicine, Seoul, Korea; ^4^ Department of Pathology, Seoul National University Bundang Hospital, Seongnam, Korea

**Keywords:** EPCAM, immunohistochemistry, colorectal cancer, lynch syndrome, prognosis, Pathology Section

## Abstract

We aimed to comprehensively investigate the clinicopathologic and molecular implications of altered epithelial cell adhesion molecule (EPCAM) expression in colorectal carcinoma (CRC). EPCAM immunohistochemical expression, *EPCAM* 3′ end deletion, *EPCAM* promoter methylation, microsatellite instability (MSI), and the CpG island methylator phenotype (CIMP) were analyzed in large cohorts of human CRCs. Among 218 MSI-high CRCs, complete loss (CL) of EPCAM expression was observed in two cases, both of which displayed MSH2 deficiency and *EPCAM* 3′ deletion. Thirty-one of the 218 MSI-high CRCs demonstrated the partial loss (PL) of EPCAM expression without *EPCAM* deletion or methylation and were correlated with CIMP-high and poor disease-free survival. Histologically, foci exhibiting EPCAM loss in EPCAM-PL tumors were dominantly distributed in poorly differentiated tumor components and/or in the invasive tumor front. The implications of EPCAM-PL were further validated in a consecutive series of 726 CRCs. EPCAM-PL (*n* = 50; 6.9%) was also associated with CIMP-high and adverse pathologic factors and was confirmed to be an independent poor prognostic factor in CRC (HR, 1.57; 95% CI, 1.04 to 2.39). EPCAM-CL can be used to screen for *EPCAM* deletion-induced Lynch syndrome-associated CRC, whereas EPCAM-PL can be used as an indicator of tumor aggressiveness and poor prognosis in CRC.

## INTRODUCTION

Epithelial cell adhesion molecule (EPCAM; also known as TACSTD1 or CD326) expression in carcinoma cells has significance as a potential diagnostic and therapeutic target. For example, EPCAM has frequently been investigated as a biomarker for detecting circulating or metastatic carcinoma cells and as a cancer stem cell marker in some malignancies [1]. Moreover, EPCAM-targeted antibodies and EPCAM-targeted drug delivery have been developed and tested for the treatment of carcinomas *in vitro* and *in vivo*, although evidence for the clinical efficacy and safety of EPCAM-targeted therapy in human cancer patients remains insufficient [1, 2].

Diffuse strong EPCAM protein expression is typically observed in colorectal carcinoma (CRC). However, several previous investigations have reported interesting findings regarding the loss of EPCAM expression in CRC. One finding showed that a small subset of Lynch syndrome-associated CRCs carrying germline *EPCAM* deletions may be associated with EPCAM expression loss in tumor cells [3–5]. Another finding was that the partial loss of EPCAM expression can frequently be observed in tumor budding at the invasive margin of CRCs [6, 7]. In addition, the poor prognostic effect of decreased EPCAM expression in CRCs has also been reported [7, 8]. Although these findings provide important insights into the implications of EPCAM loss in CRCs, the understanding regarding the detailed pattern of EPCAM loss and its significance in CRC remains incomplete.

As a pilot study, we previously evaluated the EPCAM expression status and its associations with clinicopathologic and molecular factors, including DNA mismatch repair (MMR) protein expression and the *MLH1* promoter methylation status, in 168 microsatellite instability-high (MSI-high) CRCs [9]. According to the previous study, the complete loss (CL) of EPCAM expression was found only in MSH2-deficient MSI-high CRCs, whereas the partial loss (PL) of EPCAM expression was dominantly found in MLH1-deficient and/or *MLH1*-methylated tumors. These findings indicate the existence of differential implications of EPCAM expression loss in CRC depending on the completeness of the loss. Moreover, this implies the potential connectivity between EPCAM-PL and the CpG island methylator phenotype (CIMP) in CRC. However, there is a lack of data clarifying this issue. Therefore, in the present study, we performed a more comprehensive analysis regarding the clinicopathologic, molecular, and prognostic implications of EPCAM loss in CRC. The three main focuses to elucidate in our study were as follows: (1) the molecular associations between EPCAM loss, germline deletion and promoter methylation of the *EPCAM* gene, and the MSI/CIMP statuses in CRC, (2) the histopathologic correlations of EPCAM loss in CRC, and (3) the prognostic significance of EPCAM loss in CRC.

## RESULTS

### Clinicopathologic and molecular implications of EPCAM loss in MSI-high CRCs

**Figure 1:**
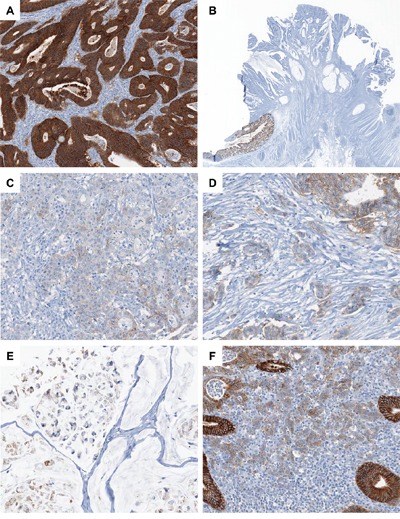
Photomicrographs of EPCAM IHC in CRC **A.** A representative case of EPCAM-intact MSI-high CRC (×100). Note the diffuse overexpression of EPCAM in the membrane and cytoplasm of the tumor cells. **B.** A representative case of EPCAM-CL MSI-high CRC (×4). Note the abrupt transition from intact EPCAM expression in the normal colonic mucosa (left lower) to the complete loss of EPCAM expression in the tumor cells (upper center). **C.** A representative case of EPCAM-PL MSI-high CRC (×200). Note the partial loss of EPCAM expression in the poorly formed tumor glands. **D.** A representative MSI-high CRC case showing partial EPCAM loss in poorly differentiated tumor cell clusters and tumor budding areas (×200). **E.** A representative MSI-high CRC case showing partial EPCAM loss in the signet ring cell component (×200). **F.** A representative MSI-high CRC case showing partial EPCAM loss in the tumor-infiltrating lymphocyte-rich invasive front area (×200).

As an initial step of our investigation, to confirm the specificity of EPCAM-CL for *EPCAM* deletion-induced MSH2 deficient Lynch syndrome-associated CRC and to find clues of the clinicopathologic significance of EPCAM-PL in CRC, we evaluated EPCAM immunohistochemistry (IHC) in a large series of primary MSI-high CRCs. Among the 218 MSI-high CRCs (discovery cohort), 2 EPCAM-CL, 31 EPCAM-PL, and 185 EPCAM-intact tumors were identified. Representative EPCAM IHC images of EPCAM-intact, EPCAM-CL, and EPCAM-PL tumors were presented in Figure 1. Molecularly, both EPCAM-CL tumors showed MSH2 expression loss and *EPCAM* gene biallelic 3′ exon deletion according to the IHC and MLPA analyses (Table 1). These molecular features indicated that these two tumors were consistent with *EPCAM* germline deletion-induced MSH2-deficient Lynch syndrome-associated CRCs. In contrast, all 31 EPCAM-PL tumors showed neither biallelic 3′ deletion nor promoter methylation of the *EPCAM* gene (Table 1). Clinicopathologically, compared with EPCAM-intact tumors, EPCAM-PL tumors were significantly associated with advanced stage (stage III/IV) (*P* = 0.001), lymph node metastasis (pN1/pN2) (*P* = 0.002), distant metastasis (*P* = 0.001), poor differentiation (*P* < 0.001), signet ring cell histology (*P* < 0.001), lymphovascular invasion (*P* = 0.01), perineural invasion (*P* = 0.02), tumor budding (*P* < 0.001), CIMP-high (*P* = 0.008), *MLH1* promoter methylation (*P* = 0.01), and wild-type *KRAS* (*P* = 0.01) in MSI-high CRCs (Table 1).

**Table 1:** EPCAM expression status-dependent clinicopathologic and molecular features in MSI-high CRCs (discovery cohort; *n* = 218)

Variables		Case No.	EPCAM-CL (*n* = 2)	EPCAM-PL (*n* = 31)	EPCAM-intact (*n* = 185)	*P*-value^a^
Age	<58 years	103	2	14 (45%)	87 (47%)	0.84
	≥58 years	115	0	17 (55%)	98 (53%)	
Gender	Male	116	1	14 (45%)	101 (55%)	0.33
	Female	102	1	17 (55%)	84 (45%)	
Tumor location	Proximal colon	141	2	21 (68%)	118 (64%)	0.71
	Distal colon	56	0	6 (19%)	50 (27%)	
	Rectum	21	0	4 (13%)	17 (9%)	
Tumor multiplicity	Solitary	196	2	28 (90%)	166 (90%)	1
	Multiple	22	0	3 (10%)	19 (10%)	
Gross tumor type	Fungating	157	1	20 (65%)	136 (74%)	0.3
	Infiltrative	61	1	11 (35%)	49 (26%)	
AJCC/UICC cancer stage	Stage I/II	141	1	12 (39%)	128 (69%)	0.001
	Stage III/IV	77	1	19 (61%)	57 (31%)	
Depth of tumor invasion (pT category)	pT1/pT2	30	1	2 (6%)	27 (15%)	0.26
	pT3/pT4	188	1	29 (94%)	158 (85%)	
Lymph node metastasis (pN category)	Absent (pN0)	145	1	13 (42%)	131 (71%)	0.002
	Present (pN1/pN2)	73	1	18 (58%)	54 (29%)	
Distant metastasis (M category)	Absent (M0)	200	2	23 (74%)	175 (95%)	0.001
	Present (M1)	18	0	8 (26%)	10 (5%)	
Tumor differentiation	WD/MD	173	2	17 (55%)	154 (83%)	<0.001
	PD	45	0	14 (45%)	31 (17%)	
Mucinous histology	Absent	93	1	11 (35%)	81 (44%)	0.38
	Present	125	1	20 (65%)	104 (56%)	
Signet ring cell histology	Absent	198	2	21 (68%)	175 (95%)	<0.001
	Present	20	0	10 (32%)	10 (5%)	
Medullary histology	Absent	211	2	29 (94%)	180 (97%)	0.26
	Present	7	0	2 (6%)	5 (3%)	
Serrated histology	Absent	194	2	28 (90%)	164 (89%)	1
	Present	24	0	3 (10%)	21 (11%)	
Lymphovascular invasion	Absent	160	2	17 (55%)	141 (76%)	0.01
	Present	58	0	14 (45%)	44 (24%)	
Perineural invasion	Absent	201	2	25 (81%)	174 (94%)	0.02
	Present	17	0	6 (19%)	11 (6%)	
Tumor budding	Absent	173	2	16 (52%)	155 (84%)	<0.001
	Present	45	0	15 (48%)	30 (16%)	
MLH1 expression	Intact	80	2	11 (35%)	67 (36%)	0.93
	Loss	138	0	20 (65%)	118 (64%)	
MSH2 expression	Intact	150	0	18 (58%)	132 (71%)	0.13
	Loss	68	2	13 (42%)	53 (29%)	
PMS2 expression	Intact	73	2	10 (32%)	61 (33%)	0.93
	Loss	145	0	21 (68%)	124 (67%)	
MSH6 expression	Intact	143	0	18 (58%)	125 (68%)	0.3
	Loss	75	2	13 (42%)	60 (32%)	
CIMP status	CIMP-high	56	0	14 (45%)	42 (23%)	0.008
	CIMP-low/negative	162	2	17 (55%)	143 (77%)	
*MLH1* promoter methylation	Methylated	64	0	15 (48%)	49 (26%)	0.01
	Unmethylated	154	2	16 (52%)	136 (74%)	
*EPCAM* promoter methylation^b^	Methylated	0	0	0 (0%)	NA	NA
	Unmethylated	33	2	31 (100%)	NA	
*EPCAM* biallelic 3′ exons deletion^b^	Present	2	2	0 (0%)	NA	NA
	Absent	31	0	31 (100%)	NA	
*KRAS* mutation^c^	Mutant	42	0	1 (3%)	41 (23%)	0.01
	Wild type	169	2	30 (97%)	137 (77%)	
*BRAF* mutation	Mutant	26	0	5 (16%)	21 (11%)	0.54
	Wild type	192	2	26 (84%)	164 (89%)	

MSI-high, microsatellite instability-high; EPCAM-CL, complete loss of EPCAM expression; EPCAM-PL, partial loss of EPCAM expression; EPCAM-intact, intact EPCAM expression; AJCC/UICC, American Joint Committee on Cancer/International Union against Cancer; WD, well differentiated; MD, moderately differentiated; PD, poorly differentiated; CIMP, CpG island methylator phenotype; NA, not applicable.

a The *p*-value was calculated using the chi-square test or Fischer's exact test. EPCAM-CL (*n* = 2) was excluded from this statistical analysis due to the extremely small sample size for this variable.

b These DNA analyses were performed only in tumors determined to have EPCAM-CL or EPCAM-PL (*n* = 33).

c *KRAS* mutation analysis was performed in 211 cases among the 218 MSI-high CRCs due to the limited amount of extracted tumor DNA.

### Histopathologic features of EPCAM loss in MSI-high CRCs

**Figure 2:**
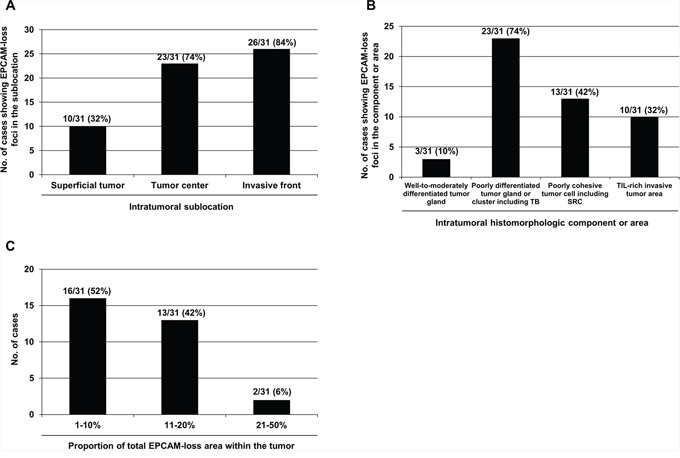
Histomorphometric analysis of the intratumoral distribution and proportion of the EPCAM-loss foci in MSI-high CRCs determined as EPCAM-PL (*n* = 31) **A.** The frequencies of EPCAM-PL tumors having EPCAM-loss foci in each intratumoral sublocation. **B.** The frequencies of EPCAM-PL tumors with EPCAM-loss foci in each intratumoral histomorphologic component or area. **C.** The frequencies of EPCAM-PL tumors according to the proportion of the total EPCAM-loss area within the whole tumor sections. Abbreviations: TB, tumor budding; SRC, signet ring cell; TIL, tumor-infiltrating lymphocyte.

We performed histomorphometric analysis of 31 EPCAM-PL MSI-high CRCs to assess the intratumoral distribution and proportion of EPCAM-loss foci. EPCAM-loss foci were more frequently observed in the invasive front (84%) than in the tumor center (74%) and superficial tumor (32%) areas (Figure 2A). Characteristically, EPCAM-loss foci were localized in poorly differentiated tumor glands or clusters (including tumor budding) (74%), poorly cohesive tumor cells (including signet ring cells) (42%), and tumor-infiltrating lymphocyte-rich invasive borders (32%) (Figure 2B). Most EPCAM-PL tumors (94%) showed a total EPCAM-loss area of less than 20% within the tumor (Figure 2C).

### Prognostic significance of EPCAM loss in MSI-high CRCs

**Figure 3:**
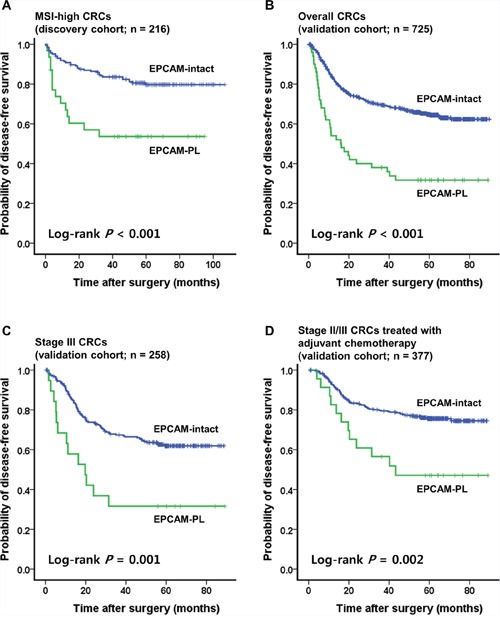
Kaplan-Meier survival analysis **A.** Significant differences of the DFS according to the EPCAM expression status in 216 patients with MSI-high CRC (discovery cohort). **B.** Significant differences of the DFS according to the EPCAM expression status in 725 patients with CRC (validation cohort). **C.** In the stage-stratified survival analysis using the validation cohort, significant differences of the DFS according to the EPCAM expression status were also maintained in patients with stage III CRC (*n* = 258). **D.** In the treatment-stratified survival analysis using the validation cohort, significant differences of the DFS according to the EPCAM expression status were also maintained in patients with stage III or high-risk stage II CRC treated with 5-fluorouracil-based adjuvant chemotherapy (*n* = 377). In all Kaplan-Meier survival analyses, EPCAM-CL cases (*n* = 2 in the discovery cohort and *n* = 1 in the validation cohort) were excluded due to the extremely small sample size.

In the Kaplan-Meier survival analysis, EPCAM-PL tumors were significantly associated with poor disease-free survival (DFS) in MSI-high CRCs compared with EPCAM-intact tumors (log-rank *P* < 0.001; Figure 3A).

### Validation of the implications of EPCAM loss in overall CRCs

To validate the clinicopathologic and prognostic implications of EPCAM-PL in overall CRCs, EPCAM IHC was performed and evaluated in a consecutive series of 726 primary CRCs. Among the 726 CRCs (validation cohort), EPCAM-CL was detected in only one tumor; this case overlapped with one of the two EPCAM-CL MSI-high tumors in the discovery cohort and also demonstrated both MSH2 loss and *EPCAM* germline deletion in repeated evaluations (Table 2). EPCAM-PL tumors were found in 50 of 726 CRCs (6.9%) and were significantly correlated with proximal tumor location (*P* = 0.001), infiltrative tumor type (*P* < 0.001), advanced stage (stage III/IV) (*P* = 0.001), deep invasion of the primary tumor (pT3/pT4) (*P* = 0.02), nodal metastasis (pN1/pN2) (*P* = 0.002), distant metastasis (*P* < 0.001), poor differentiation (*P* < 0.001), signet ring cell histology (*P* = 0.005), medullary histology (*P* = 0.003), serrated histology (*P* = 0.02), lymphovascular invasion (*P* < 0.001), perineural invasion (*P* < 0.001), tumor budding (*P* < 0.001), MSI-high (*P* = 0.03), CIMP-high (*P* < 0.001), *MLH1* methylation (*P* = 0.03), and *BRAF* mutations (*P* = 0.01) in CRCs compared with EPCAM-intact tumors (Table 2). In the Kaplan-Meier survival analysis, patients with EPCAM-PL CRC showed worse DFS than patients with EPCAM-intact CRC (log-rank *P* < 0.001; Figure 3B). This prognostic significance of EPCAM-PL was maintained in stage III CRCs (*n* = 258) and in stage II/III CRCs treated with 5-fluorouracil-based adjuvant chemotherapy (*n* = 377) (log-rank *P* = 0.001 and *P* = 0.002, respectively; Figures 3C, 3D). In the multivariate survival analysis, EPCAM-PL was proven to be an independent poor prognostic factor in CRC (hazard ratio, 1.57; 95% confidence interval, 1.04 to 2.39; *P* = 0.03; Table 3).

**Table 2:** EPCAM expression status-dependent clinicopathologic and molecular features in overall CRCs (validation cohort; *n* = 726)

Variables		Case No.	EPCAM-CL (*n* = 1)	EPCAM-PL (*n* = 50)	EPCAM-intact (*n* = 675)	*P*-value^a^
Age	<60 years	307	1	23 (46%)	284 (42%)	0.58
	≥60 years	419	0	27 (54%)	391 (58%)	
Gender	Male	441	0	27 (54%)	414 (61%)	0.3
	Female	285	1	23 (46%)	261 (39%)	
Tumor location	Proximal colon	198	1	25 (50%)	172 (26%)	0.001
	Distal colon	362	0	15 (30%)	347 (51%)	
	Rectum	166	0	10 (20%)	156 (23%)	
Gross tumor type	Fungating	476	0	21 (42%)	454 (67%)	<0.001
	Infiltrative	250	1	29 (58%)	221 (33%)	
AJCC/UICC cancer stage	Stage I/II	346	0	13 (26%)	333 (49%)	0.001
	Stage III/IV	380	1	37 (74%)	342 (51%)	
Depth of tumor invasion (pT category)	pT1/pT2	136	0	3 (6%)	133 (20%)	0.01
	pT3/pT4	590	1	47 (94%)	542 (80%)	
Lymph node metastasis (pN category)	Absent (pN0)	371	0	15 (30%)	356 (53%)	0.002
	Present (pN1/pN2)	355	1	35 (70%)	319 (47%)	
Distant metastasis (M category)	Absent (M0)	604	1	32 (64%)	571 (85%)	<0.001
	Present (M1)	122	0	18 (36%)	104 (15%)	
Tumor differentiation	WD/MD	698	1	38 (76%)	659 (98%)	<0.001
	PD	28	0	12 (24%)	16 (2%)	
Mucinous histology	Absent	639	0	40 (80%)	598 (89%)	0.07
	Present	87	1	10 (20%)	77 (11%)	
Signet ring cell histology	Absent	720	1	47 (94%)	672 (100%)	0.005
	Present	6	0	3 (6%)	3 (0%)	
Medullary histology	Absent	719	1	47 (94%)	671 (100%)	0.003
	Present	5	0	3 (6%)	2 (0%)	
Serrated histology	Absent	684	1	43 (86%)	640 (95%)	0.02
	Present	42	0	7 (14%)	35 (5%)	
Lymphovascular invasion	Absent	404	1	12 (24%)	392 (58%)	<0.001
	Present	322	0	38 (76%)	283 (42%)	
Perineural invasion	Absent	553	1	24 (48%)	528 (78%)	<0.001
	Present	173	0	26 (52%)	147 (22%)	
Tumor budding	Absent	221	1	4 (8%)	217 (32%)	<0.001
	Present	505	0	46 (92%)	458 (68%)	
MSI status	MSI-high	63	1	9 (18%)	54 (8%)	0.03
	MSI-low/MSS	663	0	41 (82%)	621 (92%)	
CIMP status	CIMP-high	46	0	14 (28%)	32 (5%)	<0.001
	CIMP-low/negative	680	1	36 (72%)	643 (95%)	
*MLH1* promoter methylation	Methylated	26	0	5 (10%)	21 (3%)	0.03
	Unmethylated	700	1	45 (90%)	654 (97%)	
*EPCAM* promoter methylation^b^	Methylated	0	0	0 (0%)	NA	NA
	Unmethylated	51	1	50 (100%)	NA	
*EPCAM* biallelic 3′ exons deletion^b^	Present	2	1	0 (0%)	NA	NA
	Absent	31	0	50 (100%)	NA	
*KRAS* mutation^c^	Mutant	180	0	14 (29%)	165 (26%)	0.68
	Wild type	507	1	35 (71%)	472 (74%)	
*BRAF* mutation^d^	Mutant	39	0	7 (14%)	32 (5%)	0.01
	Wild type	681	1	43 (86%)	637 (95%)	

EPCAM-CL, complete loss of EPCAM expression; EPCAM-PL, partial loss of EPCAM expression; EPCAM-intact, intact EPCAM expression; AJCC/UICC, American Joint Committee on Cancer/International Union against Cancer; WD, well differentiated; MD, moderately differentiated; PD, poorly differentiated; MSI, microsatellite instability; MSS, microsatellite stable; CIMP, CpG island methylator phenotype

a The *p*-value was calculated using the chi-square test or Fischer's exact test. EPCAM-CL (*n* = 1) was excluded from this statistical analysis due to the extremely small sample size for this variable.

b These DNA analyses were performed only in tumors determined to have EPCAM-CL or EPCAM-PL (*n* = 51).

c *KRAS* mutation analysis was performed in 687 cases among the 726 CRCs due to the limited amount of extracted tumor DNA.

d *BRAF* mutation analysis was performed in 720 cases among the 726 CRCs due to the limited amount of extracted tumor DNA.

**Table 3:** Univariate and multivariate survival analyses in CRCs (validation cohort; *n* = 725)^a^

Variables	*n*	Univariate analysis	*P*-value	Multivariate analysis^b^	*P*-value
		H.R. (95% C.I.)		H.R. (95% C.I.)	
EPCAM expression status					
EPCAM-intact	675	Reference		Reference	
EPCAM-PL	50	2.8 (1.95–4.01)	<0.001	1.57 (1.04–2.39)	0.03
AJCC/UICC cancer stage					
Stage I/II	346	Reference		Reference	
Stage III/IV	379	4.54 (3.38–6.09)	<0.001	3.18 (2.3–4.39)	<0.001
Tumor differentiation					
WD/MD	697	Reference		Reference	
PD	28	3.4 (2.15–5.37)	<0.001	1.62 (0.95–2.76)	0.08
Lymphovascular invasion					
Absent	404	Reference		Reference	
Present	321	2.62 (2.04–3.36)	<0.001	1.27 (0.96–1.68)	0.09
Perineural invasion					
Absent	552	Reference		Reference	
Present	173	3.19 (2.49–4.08)	<0.001	1.9 (1.45–2.48)	<0.001
Tumor budding					
Absent	221	Reference		Reference	
Present	504	2.13 (1.56–2.89)	<0.001	1.29 (0.93–1.78)	0.13
CIMP status					
CIMP-low/negative	679	Reference		Reference	
CIMP-high	46	1.81 (1.19–2.76)	0.006	1.08 (0.68–1.72)	0.73

H.R., Cox hazard ratio; 95% C.I., 95% confidence interval of H.R.; EPCAM-intact, intact EPCAM expression; EPCAM-PL, partial loss of EPCAM expression; AJCC/UICC, American Joint Committee on Cancer/International Union against Cancer; WD, well differentiated; MD, moderately differentiated; PD, poorly differentiated; CIMP, CpG island methylator phenotype

a EPCAM-CL (*n* = 1) was excluded from these Cox proportional hazards regression model-based survival analyses due to the extremely small sample size for this variable.

b Variables statistically significant in the univariate analysis were entered into the multivariate analysis.

Subgroup analyses focusing on the implications of EPCAM loss in early stage tumors were also conducted. Among the validation cohort samples, there were 27 pT1 CRCs and 235 stage II CRCs. All of the 27 pT1 cases were determined to have the EPCAM-intact phenotype without EPCAM loss. The EPCAM expression status and its clinicopathologic and molecular associations in 235 stage II CRCs were summarized in Supplementary Table S1. The only significant finding in stage II CRCs was that EPCAM-PL tumors were associated with frequent lymphovascular invasion (*P* = 0.014; Supplementary Table S1). In the Kaplan-Meier survival analysis, there was no significant difference in the survival between the EPCAM-PL and EPCAM-intact tumors in stage II CRCs (log rank *P* = 0.429; Supplementary Figure S1).

### Evaluation of EPCAM IHC in pre-operative biopsy tissues and metastatic lesions

**Figure 4:**
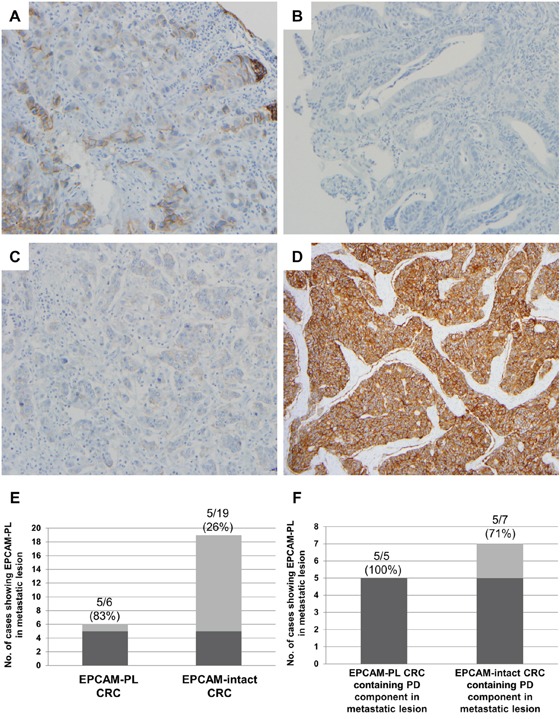
EPCAM IHC in pre-operative biopsy tissues and resected metastatic lesions **A.** A representative photomicrograph of the EPCAM-loss foci in pre-operative biopsy tissue from an EPCAM-PL CRC case (×200). **B.** A representative photomicrograph of the complete loss of EPCAM expression in pre-operative biopsy tissue from an EPCAM-CL CRC case (×200). **C.** A representative photomicrograph of the EPCAM-loss foci in a liver metastatic lesion from an EPCAM-PL CRC case (×200). Note the negative staining pattern in the poorly differentiated tumor clusters. **D.** A representative photomicrograph of intact EPCAM expression in a liver metastatic lesion from an EPCAM-intact CRC case (×100). Note the moderately differentiated tumor glands without a poorly differentiated tumor component. **E.** The frequency of EPCAM-PL in metastatic lesions from EPCAM-PL CRCs and EPCAM-intact CRCs. **F.** The frequency of EPCAM-PL in metastatic lesions containing a poorly differentiated tumor component.

Finally, the EPCAM expression status was evaluated in pre-operative biopsy tissues of EPCAM-CL and EPCAM-PL cases and in distant metastatic lesions of stage IV CRCs. Among the 51 EPCAM-loss CRCs (50 EPCAM-PL tumors and 1 EPCAM-CL tumor; validation cohort), the corresponding pre-operative biopsy tissues were available for a total of 25 cases (24 EPCAM-PL tumors and 1 EPCAM-CL tumor). Among the 25 pre-operative biopsy tissues, partial EPCAM-loss foci were observed in 9 of 24 EPCAM-PL cases (38%), and complete negativity of EPCAM expression was observed in one case that had been originally determined to be an EPCAM-CL tumor with *EPCAM* germline deletion (Figures 4A and 4B).

Among the stage IV CRCs, resection specimens of distant metastatic lesions of 6 EPCAM-PL CRCs and 19 EPCAM-intact CRCs were available for EPCAM IHC. The results were shown in Figures 4C–4F. Partial EPCAM-loss foci were observed in 5 of 6 (83%) metastatic lesions of EPCAM-PL cases and in 5 of 19 (26%) metastatic lesions of EPCAM-intact cases (Figure 4E). Histologically, the EPCAM-loss foci in the metastatic lesions were primarily distributed in poorly differentiated tumor clusters (Figures 4C and 4F).

## DISCUSSION

Our study successfully confirmed that EPCAM-CL is exclusively observed in *EPCAM* germline deletion-induced MSH2-deficient Lynch syndrome-associated CRCs, which is consistent with the findings of previous studies [3–5]. IHC for EPCAM, as well as for MMR proteins, is a simple and useful screening tool for the identification of *EPCAM* deletion-induced Lynch syndrome-associated CRCs and should therefore be included in standard diagnostics for Lynch syndrome. Interestingly, although we analyzed a large series of MSI-high CRCs (*n* = 218), including 68 cases of MSH2-deficient CRCs, we found very few tumors (*n* = 2) demonstrating both *EPCAM* germline deletions and EPCAM-CL. The prevalence of *EPCAM* germline deletions in Korean patients with MSH2-deficient CRC (3%) from our cohort is lower than the previously reported prevalence of *EPCAM* germline deletions in MSH2-deficient CRCs in Western countries (up to 10%) [10, 11]. This difference in the prevalence of *EPCAM* germline deletions in CRCs may be based on ethnic differences in the underlying molecular features of MSI-high CRCs, as suggested by our previous investigation [12].

The most notable result of our study is the comprehensive identification of clinicopathologic, molecular, and prognostic significance of EPCAM-PL in CRC. According to our data, EPCAM-PL was associated with an invasive front, poor differentiation, signet ring cell histology, tumor budding, lymphovascular/perineural invasion, nodal/distant metastasis, CIMP-high, and poor DFS in CRC. Because CIMP-high tumors are also closely correlated with poor differentiation, signet ring cell appearance, and adverse prognosis in MSI-high CRCs according to our previous study [13], it is reasonable that these clinicopathologic features would be similar between EPCAM-PL tumors and CIMP-high tumors.

Based on the significant correlation between EPCAM-PL and CIMP-high, we attempted to reveal the potential association of EPCAM-PL with *EPCAM* promoter methylation in CRC. However, we did not find any significant hypermethylation of promoter CpG islands of the *EPCAM* gene in all tested CRCs. Although the underlying molecular mechanism of EPCAM-PL in CRC was not fully elucidated in our study, several previous investigations provided important clues for the biological implications of EPCAM-PL in CRC. According to the study by Gosens et al., EPCAM loss was frequently observed in the invasive front and in tumor buds of CRCs. This observation can be interpreted as a reflection of the reduced cell-cell adhesion between actively invasive carcinoma cells [6]. Invasion and migration of carcinoma cells are known to be greatly dependent on the epithelial-mesenchymal transition (EMT), and tumor budding is a representative histologic component associated with the EMT in CRC [14]. Therefore, EPCAM-PL in the invasive front and tumor budding in CRC may represent molecular causes or consequences of the EMT, especially of the loss of cell adhesion, can be linked to other adverse pathologic factors such as lymphovascular/perineural invasion and nodal/distant metastasis, and can finally lead to poor prognosis.

Similar to the finding that EPCAM-PL was preferentially observed in poorly differentiated tumor components such as the tumor budding area of primary CRC tissues, the EPCAM-loss foci were predominantly detected in poorly differentiated tumor clusters of metastatic CRC lesions (Figures 4C and 4F). Interestingly, most metastatic lesions (83%) of the EPCAM-PL cases showed focal loss of EPCAM expression, whereas only 26% of the metastatic lesions of EPCAM-intact cases demonstrated the presence of EPCAM-loss foci (Figure 4E). These features suggest that EPCAM-PL CRCs are also closely associated with EPCAM-PL features in their corresponding metastatic lesions. However, in a detailed analysis, we found that the presence of EPCAM-loss foci completely corresponded with the presence of poorly differentiated tumor clusters in metastatic lesions of EPCAM-PL CRCs (Figure 4F). Moreover, among the 19 metastatic lesions of EPCAM-intact cases, only 7 metastatic lesions contained a poorly differentiated area, and among these, 5 cases showed the presence of EPCAM-loss foci (Figure 4F). Thus, it can be interpreted that the EPCAM-PL phenotype in metastatic lesions primarily depends on the presence or absence of poorly differentiated tumor components within the lesions. Similar to the reflection of the EMT in primary EPCAM-PL CRCs, EPCAM-PL in metastatic lesions may reflect the mesenchymal-epithelial transition (MET) in distant metastatic carcinomas.

In terms of the prognostic value of EPCAM expression loss, we revealed that EPCAM-PL is significantly associated with poor DFS in CRC compared with EPCAM-intact (Figure 3B) and is proven to be an independent poor prognostic factor in CRC (Table 3). Interestingly, in the stage-stratified survival analysis, the prognostic significance of EPCAM-PL was only observed in stage III CRCs (Figure 3C). Based on the fact that most patients with stage III CRC had received postoperative 5-fluorouracil-based adjuvant chemotherapy, we also performed the treatment-stratified survival analysis. As expected, EPCAM-PL was significantly associated with poor DFS in stage II/III CRCs treated with 5-fluorouracil-based adjuvant chemotherapy (Figure 3D). These findings indicate that EPCAM-PL may be associated with chemotherapy resistance in CRC. This inference can be reasonable because it was suggested that tumor budding, which is closely associated with EPCAM-PL, might be related to poor response to chemotherapy or chemoradiotherapy in CRC according to previous studies [15, 16]. Predictive value of EPCAM-PL in patients with CRC receiving chemotherapy or chemoradiotherapy should be further evaluated.

In terms of the clinical and experimental diagnostics, there are two important implications of our study results. First, the detection of EPCAM-CL CRC using pre-operative biopsy tissues can be useful, but the prediction of EPCAM-PL CRC using biopsy tissues may have limitations. According to our results, less than half of EPCAM-PL CRCs can be detected through EPCAM IHC in pre-operative biopsy tissues, whereas EPCAM-CL CRC can be easily detected in pre-operative biopsy specimen (Figures 4A and 4B). As described above, the EPCAM-loss foci in EPCAM-PL CRCs were predominantly distributed in the deep invasive front area. Therefore, the low detection rate of EPCAM-PL CRCs in the patients' biopsy tissues is a reasonable finding because most endoscopic biopsy tissues are obtained from the superficial tumor area. Next, based on our findings, the collection of circulating tumor cells should be used with caution to detect EPCAM-positive cells in patients with CRC because it can occasionally produce inaccurate results. Any metastasizing tumor cells derived from EPCAM-CL or EPCAM-PL CRC may show loss or downregulation of EPCAM expression, and in these cases, the circulating tumor cells cannot be detected although they are actually present. Therefore, we suggest that EPCAM IHC should be performed on primary CRC tissues before the EPCAM-based detection of circulating tumor cells is applied in patients with CRC.

In conclusion, EPCAM-CL is a highly specific indicator of *EPCAM* germline deletion-induced Lynch syndrome-associated CRC. Histopathologically, EPCAM-PL can be used as an indicator of actively invasive and aggressive tumor components in CRC. In addition, EPCAM-PL was proven to be an independent poor prognostic factor in CRC. Therefore, the use of EPCAM IHC in the routine histopathologic diagnosis of CRC could be widely helpful in the identification of aggressive pathologic factors, the prediction of survival and treatment outcomes in CRC, and the early detection of *EPCAM* germline deletion-induced Lynch syndrome-associated CRC.

## MATERIALS AND METHODS

### Tissue collection, DNA extraction, and MSI analysis

The retrospective collection of CRC tissue samples was completed in our previous studies [17, 18]. Our discovery cohort consisted of 218 pooled MSI-high CRCs, which originated from patients who underwent curative surgery for CRC at Seoul National University Hospital (Seoul, Korea) and Seoul National University Bundang Hospital (Seongnam, Korea) between 2004 and 2008. All of the cases were determined to have an MSI-high molecular status by DNA analysis [17]. Our validation cohort consisted of a consecutive series of 726 stage I–IV CRCs, which originated from all patients who underwent curative surgery for CRC at Seoul National University Hospital (Seoul, Korea) between 2004 and 2006 [18]. As previously described [18], rectal carcinomas receiving pre-operative neoadjuvant chemotherapy and/or radiotherapy were excluded in our study. Among all the samples, 56 cases overlapped between the discovery cohort (*n* = 218) and the validation cohort (*n* = 726). Therefore, a total of 888 CRC cases were finally included in our present study. Formalin-fixed, paraffin-embedded (FFPE) tissues of these 888 primary CRCs were retrieved from our pathology archives. The genomic DNA isolation from all the FFPE tissues was conducted as follows: tumor areas (tumor cells >70% of selected area) were microdissected using surgical blades from 10-μm-thick unstained slide tissues. These microdissected tumor tissues were digested in lysis buffer (100 mM Tris-HCl (pH 8.0), 10 mM EDTA (pH 8.0), 1 mg/ml proteinase K, and 0.05 mg/ml tRNA) and incubated at 55°C for 2 days. Subsequently, a 95°C incubation for 10 min was performed to inactivate proteinase K. The isolated tumor DNA was stored at −20°C until used for the analyses of MSI, *EPCAM* deletion, *EPCAM* methylation, CIMP, and *KRAS*/*BRAF* mutations. All of the 888 CRCs were subjected to MSI analysis using the fluorescent multiplex PCR method with five NCI recommended microsatellite markers (BAT-25, BAT-26, D5S346, D17S250, and D2S123) [19]. The MSI status of each CRC case was classified into one of the following categories: MSI-high (two or more unstable markers among the five markers), MSI-low (one unstable marker among the five markers) or microsatellite stable (no unstable marker among the five markers). The study was approved by the institutional review board at Seoul National University Hospital (H-1203-072-402).

### Clinicopathologic data collection and histopathologic analysis

The clinicopathologic data, including age, gender, tumor location, tumor multiplicity, gross tumor type, American Joint Committee on Cancer/International Union against Cancer (AJCC/UICC) cancer stage (7th edition), time of death, tumor recurrence, and last clinical follow-up for DFS data, of the 218 MSI-high CRCs and 726 CRCs were collected by reviewing the clinical and pathologic records of our hospitals. The histopathologic features of 218 MSI-high CRCs and 726 CRCs were evaluated by two experienced gastrointestinal pathologists (J.H.K. and J.M.B.). The assessed histopathologic factors included tumor differentiation, mucinous histology, signet ring cell histology, medullary histology, serrated histology, lymphovascular invasion, perineural invasion, and tumor budding. Tumor differentiation was assessed using a three-tier grading system based on the proportion of gland formation, as described in the World Health Organization (WHO) classification of tumors of the digestive system [20]. Tumor budding was defined as a single tumor cell or a cluster of <5 tumor cells at the invasive margin. Under a light microscope at ×200 magnification, the number of tumor buds was counted in the most intensive budding area. A tumor showing 5 or more buds in this area was considered tumor budding-positive [21]. In the subgroup analysis of EPCAM-PL MSI-high CRCs (*n* = 31), the histomorphologic components and areas of the tumors, including poorly differentiated glands or clusters, poorly cohesive cells, and tumor-infiltrating lymphocyte-rich invasive margins, were assessed on EPCAM-immunostained tissue slides.

### Immunohistochemistry

Tissue microarray (TMA) blocks of the 218 MSI-high CRCs and 726 CRCs were constructed as previously described [17]. In detail, three representative tumor areas on formalin-fixed, paraffin-embedded tissue blocks of each CRC case were selected and extracted as TMA cores (2 mm in diameter). Immunostaining for MMR proteins, including MLH1, MSH2, MSH6, and PMS2, and the subsequent analyses were performed for all 218 MSI-high CRC cases as previously described [17]. EPCAM IHC using a Ber-EP4 antibody (Ventana Medical Systems, Tucson, AZ, USA) was conducted on the TMA blocks of the 218 MSI-high CRCs and 726 CRCs as previously described [9]. An automated immunostaining technique using the BenchMark XT immunostainer (Ventana Medical Systems) was applied for all IHC procedures in this study. The EPCAM expression status of each CRC case was classified into one of the three categories: (1) complete loss of EPCAM expression (EPCAM-CL), (2) partial loss of EPCAM expression (EPCAM-PL), and (3) intact EPCAM expression (EPCAM-intact). The normal EPCAM expression pattern in tumor cells is mainly membranous staining with occasional combined cytoplasmic staining. EPCAM-CL was defined as a status showing negative EPCAM staining in 100% of tumor cells included in the TMA cores of the CRC case. EPCAM-PL was defined as a status showing negative EPCAM staining in 5% to 99% of tumor cells included in the TMA cores of the CRC case. EPCAM-intact was defined as a status showing negative EPCAM staining in less than 5% of tumor cells included in the TMA cores of the CRC case. All CRC cases determined to be EPCAM-CL or EPCAM-PL on TMA sections were repeatedly immunostained and re-evaluated on the corresponding whole tissue slides (at least two representative tumor sections) to confirm the true EPCAM-CL status in EPCAM-CL cases and to analyze the intratumoral distribution patterns of EPCAM-loss foci in EPCAM-PL cases. To further analyze the EPCAM expression status in pre-operative biopsy tissues and metastatic lesions, 25 endoscopic biopsy tissues from EPCAM-PL (*n* = 24) and EPCAM-CL (*n* = 1) cases, and 25 surgically resected metastatic lesion tissues from EPCAM-PL (*n* = 6) and EPCAM-intact (*n* = 19) cases were also stained with the Ber-EP4 antibody. EPCAM IHC was independently assessed by two pathologists (J.H.K. and J.M.B.). Conflicting assessments between the two pathologists were reviewed and discussed, and a consensus was reached.

### Multiplex ligation-dependent probe amplification

Using isolated genomic DNA samples of all EPCAM-CL and EPCAM-PL CRC cases, biallelic 3′ end deletions in the *EPCAM* gene were analyzed using the multiplex ligation-dependent probe amplification (MLPA) method, as previously described [4, 5]. MLPA was performed using the SALSA MLPA kit P072-C1 (MRC-Holland, Amsterdam, The Netherlands), which can detect large deletions in the *EPCAM* gene using probes for exons 3, 8, and 9 of the *EPCAM* gene, according to the manufacturer's protocol. MLPA data analysis based on peak area quantification to determine deletions in the 3′ exons of the *EPCAM* gene in tumor DNA samples was conducted according to the previous study by Huth et al. [4]. First, intra-sample and inter-sample normalization was performed. Subsequently, if probe ratios <0.3 were detected in probes corresponding to *EPCAM* 3′ exons, then the tumor was determined to have *EPCAM* biallelic 3′ end deletion. The MLPA analysis in EPCAM-CL/PL tumors was independently repeated twice to confirm the *EPCAM* deletions.

### DNA methylation analysis

DNA analysis for determination of the CIMP and *EPCAM* promoter methylation statuses was performed as previously described [17, 22]. Sodium bisulphite modification of genomic DNA samples extracted from the 218 MSI-high CRC tissues and 726 CRC tissues was conducted. Subsequently, the quantitative measurement of the promoter CpG island methylation of eight CIMP marker genes (*MLH1, NEUROG1, CRABP1, CACNA1G, CDKN2A* (*p16*)*, IGF2, SOCS1*, and *RUNX3*) and the *EPCAM* gene was performed using the methylation-specific real-time PCR method (MethyLight assay). CIMP analysis was conducted in all the CRC samples included in this study, whereas *EPCAM* methylation analysis was carried out only in the EPCAM-CL or EPCAM-PL CRC cases. The primers and probes used for the *EPCAM* methylation assay were designed according to a previous study by Spizzo et al.: forward primer, 5′-CACACCTACCCGACCTAACGA-3′; reverse primer, 5′-AATTTTCGGGCGGTGATTTA-3′; probe, 5′-CCCTTCCCGAAACTACTCACCTCTAACCG-3′ [22]. A CIMP-high tumor was identified as having five or more hypermethylated markers, a CIMP-low tumor was identified as having one to four hypermethylated markers, and a CIMP-negative tumor was identified as having no hypermethylated marker. A hypermethylated CpG island locus was defined when the percentage of the methylated reference (PMR) value was >4. The MethyLight assay for each CIMP marker gene and *EPCAM* gene was independently repeated three times, and the final determination of the promoter hypermethylation of each gene was made when a PMR value >4 was detected in at least two of three experiments. MethyLight-based quantitative DNA methylation analysis and the CIMP determination markers and criteria were validated through our and others' previous studies [23–26].

### *KRAS*/*BRAF* mutation analysis

*KRAS/BRAF* mutation analysis was conducted as previously described [17]. Using PCR-restriction fragment length polymorphism and direct sequencing techniques, *KRAS* codons 12 and 13 and *BRAF* codon 600 mutations were detected. Among the 218 MSI-high CRCs, 7 samples were excluded from the *KRAS* mutation analysis due to an insufficient amount of extracted DNA. Among the 726 CRCs, 39 and 6 samples were excluded from the *KRAS* and *BRAF* mutation analyses, respectively, due to an insufficient amount of extracted DNA.

### Statistical analysis

All statistical analyses in this study were performed using IBM SPSS Statistics version 20 (Armonk, NY, USA). Comparisons of the categorical variables were conducted using the chi-square test or Fisher's exact test. The DFS rates were analyzed using the Kaplan-Meier method with the log-rank test. To identify independent prognostic factors, univariate and multivariate survival analyses were performed using the Cox proportional hazards regression model. Parameters statistically significant in the univariate analysis were entered into the multivariate analysis. EPCAM-CL cases were excluded from chi-square test, Fisher's exact test, and survival analysis due to the extremely small sample size for this variable. All *P* values were two-sided, and statistical significance was determined at *P* < 0.05.

## SUPPLEMENTARY FIGURE AND TABLE


