# Supplementary material for: Clinicopathologic, molecular, and prognostic implications of the loss of EPCAM expression in colorectal carcinoma
Source: Oncotarget. 2015 Oct 30;7(12):13372–87. doi: 10.18632/oncotarget.5618 (PMC4924648; doi:10.18632/oncotarget.5618)
Supplement: Supplementary file 1 [file oncotarget-07-13372-s001.pdf]

SUPPLEMENTARY FIGURE AND TABLE

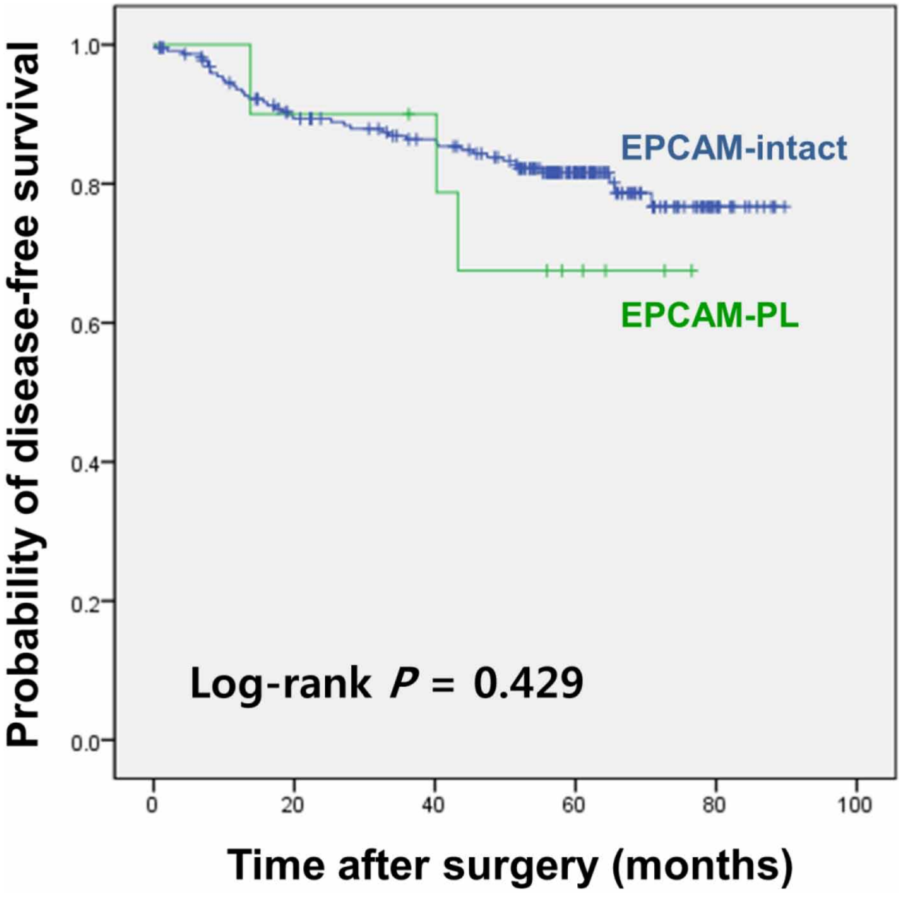

Supplementary Figure S1: Kaplan-Meier survival analysis in stage II CRCs (validation cohort; *n* = 235).

Supplementary Table S1: EPCAM expression status-dependent clinicopathologic and molecular features in stage II CRCs (validation cohort; *n* = 235)

Variables		Case No.	EPCAM-PL (n = 10)	EPCAM-intact (n = 225)	*P*-value
Age	<60 years	87	5 (50%)	82 (36%)	0.506
	≥60 years	148	5 (50%)	143 (64%)	
Gender	Male	138	3 (30%)	135 (60%)	0.097
	Female	97	7 (70%)	90 (40%)	
Tumor location	Proximal	80	5 (50%)	75 (33%)	0.311
	Distal	120	5 (50%)	115 (51%)	
	Rectum	35	0 (0%)	35 (16%)	

(*Continued*)

**Variables**		**Case No.**	**EPCAM-PL (*n* = 10)**	**EPCAM-intact (*n* = 225)**	***P*-value**
Gross tumor type	Fungating	163	5 (50%)	158 (70%)	0.18
	Infiltrative	72	5 (50%)	67 (30%)	
Tumor differentiation	WD/MD	234	10 (100%)	224 (100%)	1
	PD	1	0 (0%)	1 (0%)	
Mucinous histology	Absent	206	9 (90%)	197 (88%)	1
	Present	29	1 (10%)	28 (12%)	
Signet ring cell histology	Absent	235	10 (100%)	225 (100%)	NA
	Present	0	0 (0%)	0 (0%)	
Medullary histology	Absent	233	10 (100%)	223 (100%)	1
	Present	1	0 (0%)	1 (0%)	
Serrated histology	Absent	225	8 (80%)	217 (96%)	0.061
	Present	10	2 (20%)	8 (4%)	
Lymphovascular invasion	Absent	179	4 (40%)	175 (78%)	0.014
	Present	56	6 (60%)	50 (22%)	
Perineural invasion	Absent	203	7 (70%)	196 (87%)	0.141
	Present	32	3 (30%)	29 (13%)	
Tumor budding	Absent	86	3 (30%)	83 (37%)	0.75
	Present	149	7 (70%)	142 (63%)	
MSI status	MSI-high	29	2 (20%)	27 (12%)	0.356
	MSI-low/MSS	206	8 (80%)	198 (88%)	
CIMP status	CIMP-high	12	0 (0%)	12 (5%)	1
	CIMP-low/ negative	223	10 (100%)	213 (95%)	
MLH1 promoter methylation	Methylated	9	0 (0%)	9 (4%)	1
	Unmethylated	226	10 (100%)	216 (96%)	
KRAS mutation	Mutant	65	3 (33%)	62 (29%)	0.72
	Wild type	160	6 (67%)	154 (71%)	
BRAF mutation	Mutant	14	2 (20%)	12 (5%)	0.114
	Wild type	219	8 (80%)	211 (95%)	

EPCAM-PL, partial loss of EPCAM expression; EPCAM-intact, intact EPCAM expression; WD, well differentiated; MD, moderately differentiated; PD, poorly differentiated; NA, not applicable; MSI, microsatellite instability; MSS, microsatellite stable; CIMP, CpG island methylator phenotype
